# Vitamin B_12_: Unique Metalorganic Compounds and the Most Complex Vitamins

**DOI:** 10.3390/molecules15053228

**Published:** 2010-04-30

**Authors:** Lucio Randaccio, Silvano Geremia, Nicola Demitri, Jochen Wuerges

**Affiliations:** Centre of Excellence in Biocrystallography, Department of Chemical Sciences, University of Trieste, Via L. Giorgieri 1, 34127 Trieste, Italy

**Keywords:** B_12_ structure-function, B_12_ enzymes, cobalamins, vitamin B_12_, B_12_ transport proteins

## Abstract

The chemistry and biochemistry of the vitamin B_12_ compounds (cobalamins, XCbl) are described, with particular emphasis on their structural aspects and their relationships with properties and function. A brief history of B_12_, reveals how much the effort of chemists, biochemists and crystallographers have contributed in the past to understand the basic properties of this very complex vitamin. The properties of the two cobalamins, the two important B_12_ cofactors Ado- and MeCbl are described, with particular emphasis on how the Co-C bond cleavage is involved in the enzymatic mechanisms. The main structural features of cobalamins are described, with particular reference to the axial fragment. The structure/property relationships in cobalamins are summarized. The recent studies on base-off/base-on equilibrium are emphasized for their relevance to the mode of binding of the cofactor to the protein scaffold. The absorption, transport and cellular uptake of cobalamins and the structure of the B_12_ transport proteins, IF and TC, in mammals are reviewed. The B_12_ transport in bacteria and the structure of the so far determined proteins are briefly described. The currently accepted mechanisms for the catalytic cycles of the AdoCbl and MeCbl enzymes are reported. The structure and function of B_12_ enzymes, particularly the important mammalian enzymes methyltransferase (MetH) and methyl-malonyl-coenzymeA mutase (MMCM), are described and briefly discussed. Since fast proliferating cells require higher amount of vitamin B_12_ than that required by normal cells, the study of B_12_ conjugates as targeting agents has recently gained importance. Bioconjugates have been studied as potential agents for delivering radioisotopes and NMR probes or as various cytotoxic agents towards cancer cells in humans and the most recent studies are described. Specifically, functionalized bioconjugates are used as “Trojan horses” to carry into the cell the appropriate antitumour or diagnostic label. Possible future developments of B_12_ work are summarized.

## 1. Introduction

**Figure 1:**
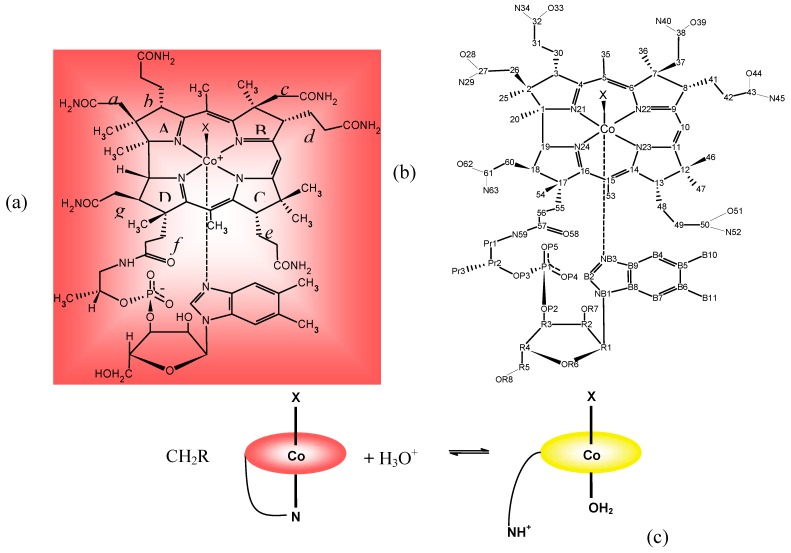
(a) Structural formulas of vitamin B_12_ (X=CN) and of the biologically active cobalamins (X=Ado, X=CH_3_); (b) conventional atoms nomenclature for cobalamins; (c) base-on form (left side) and base-off form (right side); only the number or the symbol are indicated for the carbon atoms.

The first observation of pernicious anaemia occurred in 1824, after which it took about one hundred years before Whipple, Minot and Murphy discovered that after treatment with liver “…all the patients showed a prompt, rapid, and distinct remission of their anemia…” and that liver must contain an anti-pernicious anaemia factor [1]. In 1934 they shared the Nobel Prize for Medicine and Physiology. This period can be described as the early medicinal era of vitamin B_12_. After the Second World War, Folkers at Merck, Sharp and Dohme and, soon afterwards, Smith at Glaxo, isolated and crystallized that factor, which was named vitamin B_12_ [1], now also called cyanocobalamin (CNCbl). In 1955, the elucidation of the X-ray structure of vitamin B_12_ was finally accomplished by Dorothy Hodgkin [2], to whom the Nobel Prize for Chemistry was awarded in 1964. The compound was shown to be an octahedral cobalt compound as shown in Figure 1a. 

In 1972, Woodward and Eschenmoser reported the total synthesis of vitamin B_12_ as a result of a collaborative effort involving more than one hundred scientists over eleven years [3]. In the meantime, it was found that cyanocobalamin was an artefact due to the method of isolation from liver and that the biologically important cofactors were the yellow-orange cobalamin, 5’-deoxy-5’-adenosylcobalamin, often called B_12_ coenzyme or adenosylcobalamin (AdoCbl), and the red methylcobalamin (MeCbl). Since these early years, thousands of articles have been published describing B_12_ chemistry, biochemistry and enzymology [4,5,6,7,8], giving rise to the chemical and biological era of vitamin B_12_. This period also includes the complete elucidation of the biosynthetic pathway to vitamin B_12_ in aerobic [9] and anaerobic [10] microorganisms as well as the recent X-ray structural determination of several B_12_-based enzymes [11] which play a basic role in B_12_ enzymology [10]. Finally, the recent studies [12] and determination of the X-ray structure of the B_12_ transport proteins in mammals [13,14] have provided the basis for designing Cbl-based bioconjugates [15,16,17] for imaging tumours; as well as for selected delivery of anti-tumour agents to malignant cells. This will possibly give rise to a new medicinal era of vitamin B_12_.

In cobalamins (Cbl), the Co atom is equatorially coordinated by the corrin ligand possessing seven amide side chains (*a-g*) (Figure 1a). The numbering scheme for the atoms of this complex molecular system introduced by Dorothy Hodgkin is shown in Figure 1b [2]. The corrin contains four reduced pyrrole rings, A-D, with a direct connection between rings A and D, and has a single helical sense of absolute *R*-configuration of its chiral centres at C1 and C19. Chain *f* is connected through an amide bond to a nucleotide, whose the 5,6-dimethylbenzimidazole base coordinates Co at the axial position on the α side (lower side) of the corrin macrocycle. Cobalamins with the Co in the oxidation state +3, are generally octahedral, with the axial X ligand coordinated on the β side (upper side) of the corrin ligand in the base-on form (Figure 1c, left side). Cobalamins assume the base-off form (Figure 1c, right side) upon protonation of the coordinated NB3 atom and benzimidazole is substituted by an exogenous ligand. In the Co(II) oxidation state, the cobalamin has no β axial ligand and it is generally known as cob(II)alamin or B_12r_, whereas in the Co(I) state it is presumably tetracoordinate in a base-off form and without the β axial ligand (cob(I)alamin or B_12s_). Corrinoids having structural modification with respect to cobalamins, such as a different base in the nucleotide loop or with changes in the corrin ligand, are preferentially indicated as cobamides (Cba), specifying the axial ligand(s) and the nucleotide base, if necessary [4]. The cobinamides (Cbi) are related nucleotide-free corrinoids, in which the X and Y ligands in the axial position are specified by α and β, according to their position on the two sides of the corrin ring.

## 2. The B_12_ Cofactors

As already stressed, CNCbl is not known to have a direct biological role, whereas MeCbl (X = CH_3_ in Figure 1a) and AdoCbl (X = 5’-deoxyadenosylcobalamin in Figure 1a) are cofactors of several enzymes, which are the only ones thus far known whose cofactors contain a metal-carbon bond involved in the enzymatic reactions. In fact, they catalyze enzymatic reactions which involve the making and breaking of the Co-C bond of these cofactors. 

MeCbl is the cofactor of several methyltransferases, such as methionine synthase (MetH), which catalyzes methionine biosynthesis both in mammals and bacteria. Methylcorrinoids, including MeCbl, are also cofactors of enzymes participating in the carbon dioxide fixing pathway in anaerobic acetogenic bacteria [18] and methanogenic archaea [19]. The enzymatic mechanisms for the methyl transfer require the formally heterolytic cleavage and formation of the Co-Me bond often in presence of an additional cofactor, either a Zn^2+^ ion in MetH [20] or a [Fe_4_S_4_] cluster in acetogenic bacteria [18]. The cofactor is always found to bind the apoenzyme in the base-off form and, in a subset of methyltransferases, the Co is coordinated on the α side by a histidine from the protein scaffold in the so called base-off/His-on binding mode [5]. 

AdoCbl is the cofactor of several enzymes, eliminases and isomerases (or mutases), which catalyze the 1,2 shift of an H atom and an electronegative group on adjacent carbon atoms, such as L-methyl-malonyl-CoA-mutase (MMCM). MMCM catalyzes the reversible isomerisation of L-methylmalonyl into the succinyl residue in mammals and bacteria. The MMCM enzyme is the only one of the AdoCbl-based enzymes found in mammals. AdoCbl is also cofactor of the class II ribonucleotide reductase (RNR), which catalyzes the reduction of ribonucleoside tri- or diphosphates to 2′-deoxyribonucleoside tri- or diphosphates in bacteria [21]. All the AdoCbl enzymatic mechanism requires the reversible homolytic cleavage of the Co-Ado bond. The AdoCbl coenzyme is found to bind to the apoenzyme in base-on form in B_12_ eliminases (with K^+^ ion as additional cofactor) and in RNR, while the base-off/His-on binding occurs in isomerases.

Cobalamins are also found to be cofactors of enzymes in anaerobic sulphidogenic bacteria, which are able to reductively dechlorinate aliphatic and aromatic chloro-hydrocarbons [22]. These enzymes contain Fe_4_S_4_ and Fe_3_S_4_ clusters in addition to a corrinoid. In the tetrachloroethylene dehalogenase the cofactor is a novel corrinoid, the norpseudo-B_12_ [23], whose nucleotide base is adenine and the Pr3 carbon atom (Figure 1b) is substituted by an H atom. 

The most striking feature of the two coenzymes, AdoCbl and MeCbl, is the Co-C bond, which has been extensively studied, as its cleavage is involved in the intricate pathways of B_12_ metabolic functions. These studies have concerned enzymes, cobalamins and the simple model, cobaloximes [4,5,24]. 

Some ground state properties of the axial fragment in the two coenzymes are reported in Table 1. Comparison of the Co-C distances, stretching frequencies and BDE’s, reported in Table 1, indicates that in the ground state, the Co-C bond in MeCbl is stronger than that in AdoCbl. Comparison of the Co-NB3 distance indicates that Ado exerts a stronger trans influence than Me [25]. The unusually wide Co-CH_2_-C angle of 124.3° is to be noted in AdoCbl.

In principle, in alkylcobalamin (RCbl) the Co-C fission can take place by a homolytic (1) or by one of the two heterolytic mechanisms (2 and 3):
1)RCo(III)alamin → cob(II)alamin + R^−^2)RCo(III)alamin → cob(III)alamin + R^−^3)RCo(III)alamin → cob(I)alamin + R^+^

In most of these reactions the immediate products cannot be detected because they undergo further reactions. Cob(II)alamin, however, is stable and it has been structurally characterized [5]. Reactions 1 and 3 occur in anaerobic conditions. All the above reactions have been widely studied in alkylcobalamins and alkylcobaloximes [4,5,6,7,26]. Both cofactors undergo photolysis and thermolysis. AdoCbl thermolysis and photolysis occur through reaction 1, with a rate constant k = 1.16·10^-8^ s^-1^ at 30 °C, and this is favoured over heterolysis at higher temperature [6,7]. It is generally accepted that the homolysis of the Co-C bond in AdoCbl is the initial step in the AdoCbl enzymatic mechanism (reaction 1). It has been documented [6,7] that the rate constant of AdoCbl homolysis in the holoenzyme increases enormously (more than ten orders of magnitude) with respect to that found in the isolated coenzyme. The manner in which the binding of AdoCbl to the apoenzyme accomplishes this rate enhancement remains unknown, in spite of a large number of studies, and it is still the subject of research.

The heterolytic cleavage in MeCbl occurs by the transfer of the CH_3_^+^ carbocation to a suitable nucleophile, but homolysis of MeCbl has been also studied [6,7].

## 3. Relevant Structural Aspects of Cobalamins

Before analysing the relationships between properties and structure in cobalamins, some of their main structural features are summarised in this section. Only in the last decade, thanks to the use of synchrotron X-ray sources coupled to area detectors, have a good number of structures of cobalamins been reported with accuracy similar to that of high resolution structures of small molecules. Very recently, their structural features have been reviewed [25,28]

**Figure 2:**
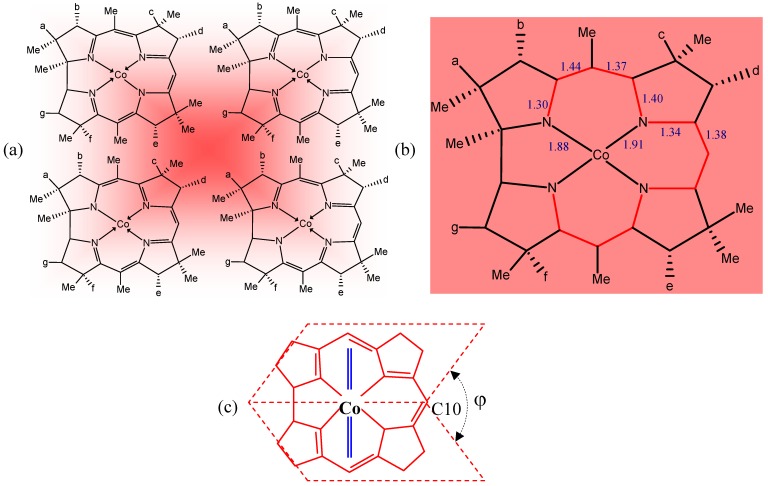
a) The main four resonance structures for the delocalized corrin system; b) mean bond lengths within the delocalised moiety of the corrin nucleus [25]; c) the folding angle ϕ. The double blue line represents the trace of the 5,6-benzimidazole group.

The internal moiety of the corrin ligand has a π delocalized system involving the N and the sp^2^ C atoms (Figure 1). The four main resonance structures of the corrin moiety are shown in Figure 2a. Accurate experimental values of the distances of the equatorial moiety indicated that they are scarcely affected by the kind of the X axial ligand. Furthermore, the Co-N distances involved in the five-membered ring are significantly shorter than the other two equatorial Co-N distances (by about 0.02 Å). 

The experimental C-C and C-N bond lengths within the delocalized moiety have a trend which reflects the approximately two-fold symmetry with respect to the axis passing through Co and C10 (Figure 1b) [25]. This trend can be fairly well interpreted on the basis of the four main resonance structures of Figure 2a. In fact, linear relationships can be found between the experimental values of the C-C and C-N distances and the corresponding bond orders, roughly derived from the four resonance structures. The trend of the C-C single bond lengths of the peripheral moiety of the corrin, which vary from 1.508 Å of the C8-C9 bond to 1.580 Å of the C1-C2 bond, reflects the different hybridization and the number of non-H substituents at the bonded C atoms (Figure 2b). The preferred mode of deformation of the corrin ligand is represented by a folding towards the X axial ligand, about an axis approximately bisecting the C1-C19 bond and passing through C10. The deformation is measured by the folding angle *φ*, which is generally calculated as the dihedral angle between the planes (Figure 2c) passing through N21, C4, C5, C6, N22, C9, C10 and C10, C11, N23, C14, C15, C16, N24 (Figure 1b). The folding angle *φ* roughly decreases with increasing Co–NB3 distance, *i.e.* with an increase in the *trans* influencing ability of X, rather than with an increase of its bulk. In fact, the steric pressure of the benzimidazole residue (indicated by the double blue line in Figure 2c) on the equatorial ligand is released when the Co-NB3 bond lengthens because of the increase in the *trans* influencing ability of X [25].

For several XCbl complexes, with X varying from H_2_O to NO, the Co-NB3 axial distance lengthens by about 0.4 Å, being 1.925(2) Å in the aquocobalamin (H_2_OCbl^+^) and 2.349 (2) Å in NOCbl, respectively. The lengthening of that bond *trans* to X reflects the increase in the electron σ-donating ability of X (electronic *trans*-influence). The alkyl groups are among the stronger *trans*-influencing ligands as compared to the “inorganic” ligand, the order of increasing *trans*-influencing ability of X donor atom being O < N < S < C. For the same X group, the lengthening of the Co-NB3 bond was found to be more enhanced with respect to cobaloximes and XCo(NH_3_)_5_. On the contrary, the Co-X distances do not vary when compared to the analogous ones in cobaloximes or in XCo(NH_3_)_5_, except when X is either a weak electron σ-donating group, such as H_2_O, or a good electron π-acceptor, such as CN^−^ [25,28]. This was interpreted as a consequence of the electronic *cis*-influence of the equatorial moiety on the axial bonds [26,28]. In alkylcobalamin the increase in bulk of the alkyl group, R, provokes a weakening of the Co-C bond (Table 1) due to the steric interaction of R with the corrin ligand (*cis*-influence). Interestingly, the X-ray structure of NOCbl has shown [29,30] that the inorganic nitroxyl ligand (with a bent Co-N-O geometry) exerts a significantly larger *trans*-influence, as measured by the length of the Co-NB3 bond, than that of an alkyl group [31] (see Section 4). However, NMR and UV-Vis spectroscopies have furnished evidence that a significant fraction, about one third, of NOCbl in solution is present in base-off form [32]. In alkylcobalamins (including CNCbl), it has been found that both Co-NB3 and Co-C distances increase when both the bulk and the electron donating ability of X increase: this trend is called inverse *trans*-influence, in contrast to the regular *trans*-influence which occurs when the Co-X shortens and the *trans* bond lengthens, as is observed in a series of cobalamins with X = ligands containing a S(sp^3^) donor [25,28]. A theoretical study by DTF calculations [33] agrees with that experimental finding. However, this study has also shown that normal *trans*-influence can be observed for substituted alkyl groups, such as C(CN)_3-n_Cl_n_ (with n = 1, 2, 3), which have similar bulk but decreasing electron withdrawing ability from n = 0 to n = 3, although these molecules have not been so far structurally characterized.

## 4. Some Structure/Properties Relationships

The first step of thermolysis and photolysis of alkylcobalamins is the homolysis of the Co-C bond, the different type of R radical leading to different final products. On the contrary, heterolysis of the Co-C bond is observed in reactions either with nucleophiles such as a cyanide (displacement reaction), the final product being the corrinoid (CN)_2_Cbl, or with electrophilic agents, such as acids [6,7].

**Scheme 1:**
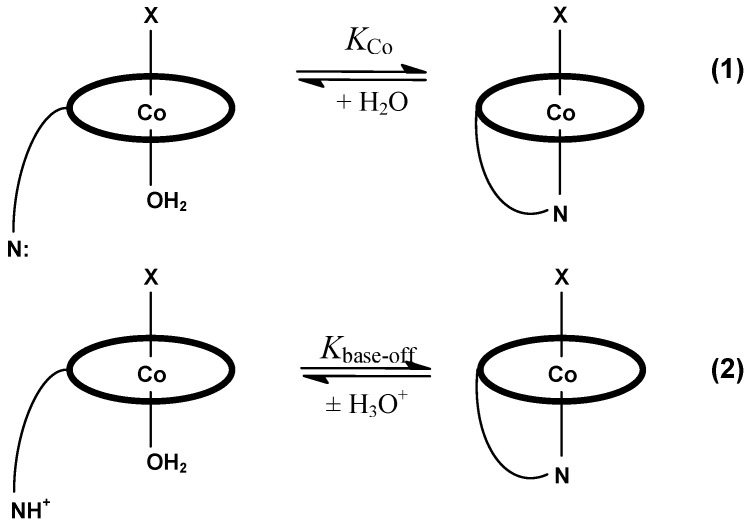
Dissociation equilibrium of the benzimidazole ligand to give the unprotonated (1) and protonate (2) base-off form of cobalamin.

In view of the finding that in several enzymes, both cofactors have been found to bind the protein in the base-off/His-on mode, the most relevant ligand substitution reaction in cobalamins is the intramolecular dissociation of the benzimidazole ligand to form the base-off form, according to equilibrium 1 (Scheme 1). It has been shown that *K*_Co_ (and consequently the apparent Δ*G*°) for reaction 1 may be calculated from the experimental values of *K*_base-off_ for equilibrium 2 (Scheme 1), measured in sufficiently strong acid solution [34]. The *K*_Co_ values obtained for a series of XCbl, for X ranging from NO to H_2_O [6,7,32,34], are reported in Table 2, together with the corresponding Δ*G*° values, the observed *K*_base-off_ and the Co-NB3 distances. 

It was observed that the values of Δ*G*° increase with the increase in the electron σ-donating ability of X. The increase in ΔG° (thermodynamic trans-influence) for the above X ligands (including the recently reported EtCbl data [31]) is linearly related to the increase in the Co-NB3 distances (Table 2) (structural trans-influence).

The correlation factor R^2^ of 0.94 for the eleven Cbl’s of Table 2, becomes 0.98 when the regression is limited to the seven cobalamins having Co-NB3 distances with e.s.d.’s ≤ 0.005 Å. It should be noted that the very recently reported Co-NB3 distance in EtCbl well fits the correlation [31]. Actually, in the first determination of NOCbl [29] a Co-NB3 distance of 2.123(5) Å was reported, which put NOCbl tremendously out of the line. Subsequently, it has was found that NOCbl was partially oxidized to O_2_NCbl and the crystals had as axial X ligand both NO and NO_2_. A new X-ray analysis has shown that in non oxidized NOCbl the Co-NB3 distance was actually 2.349(2) [30], which allowed NOCbl to fit very well the correlation. Similarly, a fairly good linear correlation is found between the ^31^P NMR chemical shifts against the Co-NB3 distances [32]. To our knowledge, the above results represent the only quantitative examples of structure/properties correlation for a complex chemical system such as cobalamins. 

The coordinated water molecule in H_2_OCbl (vitamin B_12a_) is easily displaced by several other Z ligands according to the reaction in aqueous solution:

H_2_OCbl + Z ⇌ ZCbl + H_2_O

The equilibrium constant, *K*, for the above reaction varies by several orders of magnitude when the Z ligand is varied. For example, the trend of log*K* for the following Z ligand (*K* in parentheses) is: CN^−^(14.1) >> SO_3_^2-^ (7.8) > OH^−^ (6.2) >N_3_^−^ (4.9) ~ imidazole (4.6) > I^−^ (1.5) > Cl^−^ (0.1) [35]. The trend of log*K* shows no correlation with that of the *trans*-influence. There is evidence that this order of the binding affinity of the Z ligands to cobalt, as expressed by the *K* values, is also maintained, at least qualitatively, in the cobalamins bound to protein (see Section 5.1 and 6.1.1).

In addition to the reactivity of Co at the axial position, the elevated number of functionalities available on the side chains of the corrin allows many reactions [4,5,6,7], which give a huge number of derivatives, including bioconjugates (Section 7).

## 5. Absorption, Transport and Cellular Uptake of B_12_

**Figure 3:**
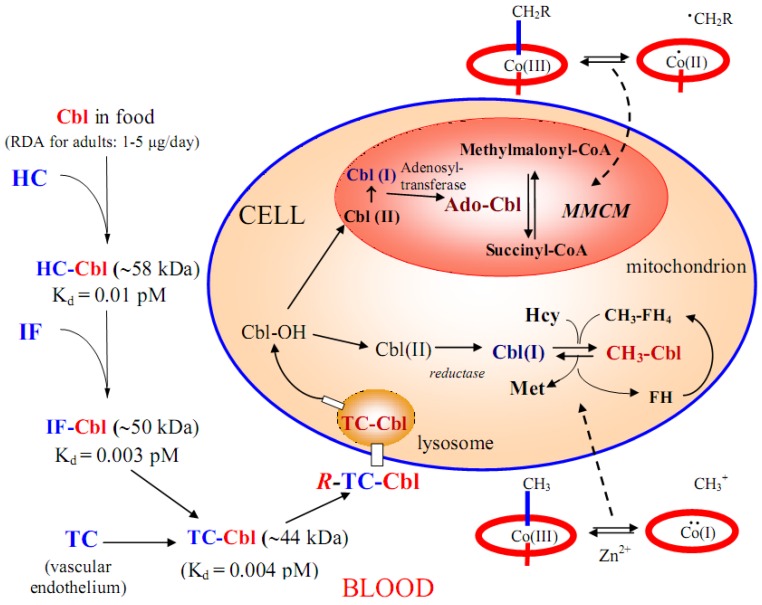
Absorption, transport and cellular uptake of cobalamins in mammals. The schematic formation and function of the two cofactors, AdoCbl and MeCbl within the cell. The homolytic and heterolytic cleavage of the Co-C bond in AdoCbl and MeCbl enzymes, respectively are evidenced. The corresponding arrow indicates where the cleavage occurs, homolysis into the mitochondrion and heterolysis into the cytoplasm.

Cobalamins cannot be synthesized by higher organisms and must be supplied with the diet. In humans, the lack of dietary Cbl or malfunctioning of absorption or of the enzymatic catalysis may provoke neurological disorders, in addition to pernicious anaemia [5]. Mammals have developed a complex pathway, sketched on the left side of Figure 3, for absorption, transportation and cellular uptake of cobalamin. This pathway involves three separate binding proteins, haptocorrin (HC), intrinsic factor (IF) and transcobalamin (TC), which form tight complexes with Cbl [36,37]. The dietary Cbl is preferentially bound to salivary HC to form the complex HC-Cbl. Pancreatic proteases in the duodenum cleave HC, Cbl is released and then binds to IF forming IF-Cbl [38]. Inside the enterocytes, the IF is degraded and the free Cbl binds to TC. The TC-Cbl complex is then released into plasma, where it is endocytosed by membrane receptors, R-TC-Cbl [39,40] (Figure 3, left side). Inside the target cells, TC-Cbl is degraded into lysosomes, releasing cobalamin molecules which are metabolized to the two cofactors: 5’-deoxyadenosyl-Cbl in mitochondrion and methyl-Cbl in cytosol (Figure 3, right side) [41]. Recent developments on the intracellular B_12_ trafficking have been recently reviewed by Banerjee *et al*. [42] The cells of bacteria, which need to import B_12_ through the cell membrane, have developed surface binding sites with very high affinities for Cbl, which is then released and internalized into the cell. The only Cbl transport system in bacteria extensively studied is that of *Escherichia coli* [43] and recently the properties and the X-ray structure of several protein components involved in this system have been reviewed [28,11].

### 5.1. B_12_ transport proteins in mammals

**Figure 4:**
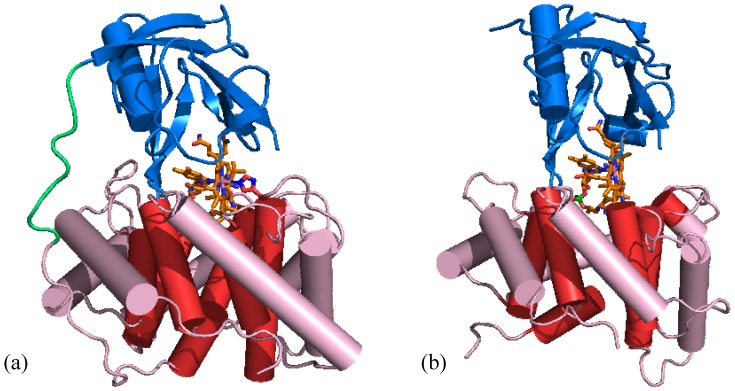
The α-β domain structure of transcobalamin (TC) and human intrinsic factor (IF). a) The two-domain structure of TC; the internal six α helices are in red and the six external ones in violet. The β domain is in blue. The loop connecting the two domains is in green. b) The two-domain structure of IF; the internal six α helices are in red and the six external ones in violet. The β domain is in blue. The figures are generated from PDB files 2BB5 (a) and 2PMV (b).

The three B_12_ transport proteins in mammals (TC, HC and IF) have similar peptidic molecular weights of about 45 kDa, as shown on the left side of Figure 3. However, HC and IF are glycosylated. Each transport protein carries a single Cbl molecule which is very tightly bound. The dissociation constants, *K*_d_, for all the three protein-Cbl complexes have very small values of about 10^-15 M^ [44], as shown on the left side of Figure 3. This is the reason why, in order to release Cbl, degradation of the transport protein occurs at each step. However, the three proteins differ in specificity for cobalamin modified analogues in the order HC << TC < IF. Compared with the explosion of X-ray structural work on B_12_ enzymes started in 1994 (see next Section), no crystallographic study of B_12_ transport proteins was reported until 2006, when the structure of human and bovine TC in complex with Cbl was reported [13]. The structure of the two TC-Cbl complexes is very similar and consists of two domains, α and β, connected by a flexible linker (Figure 4a). 

The α-domain is composed of twelve α helices arranged in a α6α6 barrel and a short 3/10 helix, whereas the β-domain consists of two β sheets and one α helix. Cbl is bound in base-on form with the corrin plane nearly perpendicular to the interface of the two domains (Figure 4). The water molecule of H_2_OCbl used in the expression protocol is displaced by the imidazole of a histidine residue from the α-domain. This binding mode of Cbl, His-on/base-on is different from either the base-on or base-off/His-on mode found in enzymes.

The X-ray structure of IF in complex with CNCbl has also been reported [14] and is very similar to that of the TC-Cbl (Figure 4b), in spite of the low sequence identity between the two transport proteins (23%), which instead show a considerable higher sequence similarity in the stretches with secondary structure. As in the TC-Cbl complex, Cbl is bound in the base-on mode. However, the axial CN group was not detected, since it was probably photolyzed during X-ray data collection and the pentacoordinated cobalt probably has oxidation state +2. On the other hand, in the IF aminoacid sequence there is no His residue available for Co binding. The difference in binding of Cbl to TC with respect to IF (and probably to HC [45]) explains why the kinetics of the binding of H_2_OCbl to TC is different from that to HC and IF [44]. In the crystal of the IF-CNCbl complex for each couple of α- and β-domain, an additional isolated α-domain was found. Furthermore, the loop joining the two α and β domains binding CNCbl was not detected (Figure 4b). These results strongly suggest that in some stage of manipulation of the sample (purification, crystallization, etc.) the two domains were cleaved. This cleavage has been studied in solution and it has been shown that the two fragments, IF_30_ and IF_20_, are still able to tightly bind CNCbl and that the formed complex, IF_30_·Cbl·IF_20_, is recognized by the IF receptor, cubilin, as the non cleaved IF-Cbl complex [46]. The X-ray structure of TC-Cbl crystallized in the presence of an excess of cyanide or sulphite ions have been reported [47]. As expected, on the basis of the binding constants of these ligands to Cbl, significantly larger than that of imidazole, the CN and sulphite ligands displace, respectively the histidine residue in TC and coordinate Co at the β axial position. Consequently, the loop containing the coordinated histidine, clearly detected in TC-Cbl, becomes too disordered to be detected on the corresponding electron density maps of TC-CNCbl and -SO_3_Cbl [47]*.* Thus, the access to the Cbl β side also becomes allowed to solvent or external ligands. In TC-Cbl, cobalamin is almost completely buried between the two domains and its solvent-accessible surface is reduced to about 7%, leaving only the ribosyl CH_2_OH group exposed to solvent. On the contrary, in TC-CNCbl and TC–SO_3_Cbl, as well as in IF-CNCbl, where the also the corrin β side is exposed to solvent, the solvent-accessible surface of Cbl is increased to 17%. This suggests that the most promising B_12_ positions to which a probe can be covalently bound through a suitable linker, without impairing the cobalamin binding to TC or IF, are the Co at the β axial side and the 5’-OH group of the ribose moiety [13,14], in agreement with previous findings in solution [48] .

### 5.2. B_12_ transport proteins in bacteria

**Figure 5:**
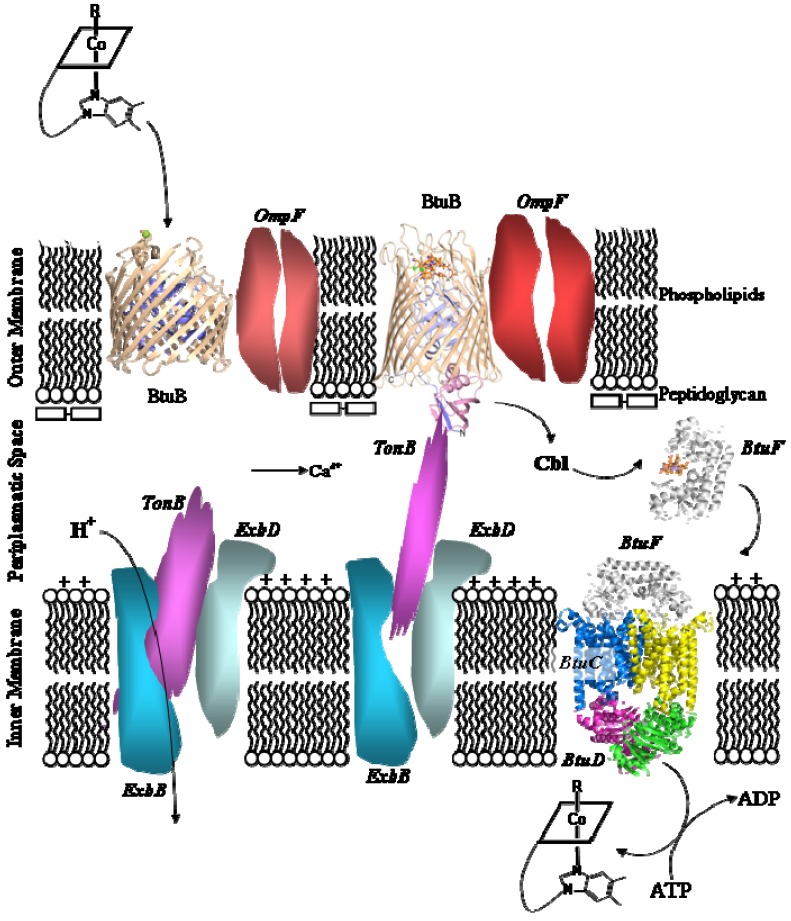
Hypothetical scheme of cobalamin uptake in *E. coli*. The proteins involved in the transport and B_12_ cellular uptake, whose X-ray structures are available, are shown by the ribbon representation for the protein moiety and by stick and ball for cobalamin. The X-ray structures of OmpF, ExbB and ExbD have not been so far determined and are indicated by a coloured form positioned into the membranes. The figures of proteins are generated from PDB files 2GUF (BtuB), 2GSK (BtuB-TonB), 1N2Z (BtuF) and 2QI9 (BtuC-BtuD-BtuF).

B_12_ transport in mammals occurs as sketched in Figure 3 (left side), whereas in *E. coli* (and other Gram-negative bacteria) a hypothetical representation of the Cbl import across the membrane [43] is sketched in Figure 5. The B_12_ transmembrane import is accomplished by the B-twelve-uptake (Btu) system, consisting of an outer membrane transporter BtuB (Figure 5, left side) and an ATP-binding cassette (ABC) transporter, BtuC_2_D_2_F, located in the inner membrane (Figure 5, right side). 

Substantially, it is proposed that Cbl is taken up by BtuB, located in the outer membrane, and then released in the periplasmatic space (Figure 5, left side). Cbl binds to BtuB with high affinity (*K*_d_ ~0.3 nM) in presence of calcium ions; depletion of the latter reduces the affinity 50-100 fold [43]. In the periplasmatic space Cbl is bound to BtuF with *K*_d_ ~0.15 nM. The BtuF-Cbl complex binds to the periplasmatic side of BtuC_2_D_2_, located in the inner membrane (Figure 5, right side), and feeds Cbl to the latter. The hydrolysis of ATP, located between the two BtuD domains powers the import of Cbl in the *E. coli* cells. The X-ray structure of several components of the Btu system, namely BtuB (in the apo form and bound to CNCbl in base-on form) [49], BtuB in complex with a C-terminal domain of TonB [50], the BtuC_2_BD_2_ component without Cbl, but with cyclotetravanadate located in the ATP binding site [51], and the periplasmatic binding protein BtuF (in the apo form and in complex with CNCbl in base-on form) [52,53] have been reported and most of them recently reviewed [11]. Very recently, the structure of the ABC transporter (BtuC_2_D_2_ in complex with ButF), without ATP and Cbl has been reported [54]. In this complex, the twofold symmetry of the two BtuC subunits observed in BtuC_2_D_2_ is lost and the translocation pathway is closed on both sides of the membrane. e.p.r. spectra are consistent with the conformation of BtuC_2_D_2_F observed in the crystal structure. All available X-ray structures are represented in Figure 5 with the usual ribbon representation for the protein moiety and with ball and stick for cobalamin. The two essential Ca^2+^ ions inside each BtuB unit are represented by green spheres.

## 6. B_12_ Dependent Enzymes

Following the first X-ray structure of the cobalamin-binding domain (Cap-Cob) of MetH in 1994 [55] and of the complete MMCM [56] in 1996, in the last ten years there has been an explosion of work on B_12_ dependent enzymes. This is due to the availability of overexpressed enzyme on the one hand, and to the successful efforts in obtaining the X-ray structure of several enzymes on the other [6]. As stressed in Section 2, these enzymes can be grouped in two broad classes, those having as cofactor MeCbl or related methylcorrinoids and those having AdoCbl. Very recently, the literature on these enzymes found in all three kingdoms of life has been extensively reviewed [8]. In this section, the metabolic role of these enzymes, particularly the only corrinoid-dependent enzymes found to act in mammals (MetH and MMCM), will be briefly described, together with their most relevant structural features. Detailed information about reactivity of the B_12_-dependent enzymes can be found in the exhaustive review by Brown in ref. [6].

### 6.1. Corrinoid-dependent methyltransferase

**Figure 6:**
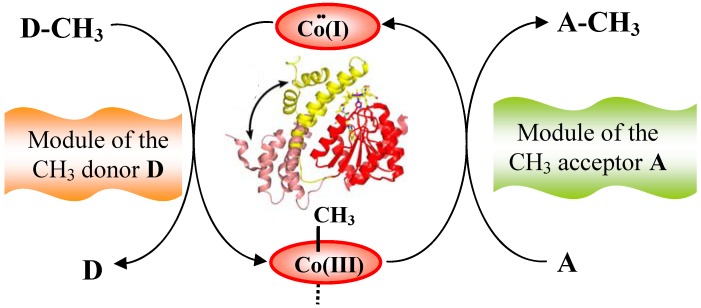
The three module representation of cobalamin methyltransferases.

The basic pattern for corrinoid methyltransferases has been elegantly presented and discussed by Rowina Matthews [8] and is shown in Figure 6. It is represented by three modules. In the central module, Co(I) of the corrinoid abstracts a methyl group from the substrate methyl-donor (D-CH_3_) present in the external module (the left side of Figure 6) and Co(III)-CH_3_ transfers its methyl to the methyl-acceptor (A) in the external module (the right side of Figure 6) to give the methyl acceptor product (A-CH_3_). Often, a cofactor such as a zinc cation is associated with one of the two external modules. Due to the ease of Co(I) to undergo oxidation to Co(II), the central module is often associated with a reactivation domain, which restores Co(III)-CH_3_ by reductive methylation. The three modules of Figure 6 may belong to the same protein chain, as in MetH, or to a system of separate proteins, as in the enzymes involved in acetogenesis and methanogenesis.

#### 6.1.1. MeCbl methionine synthase (MetH)

**Figure 7:**
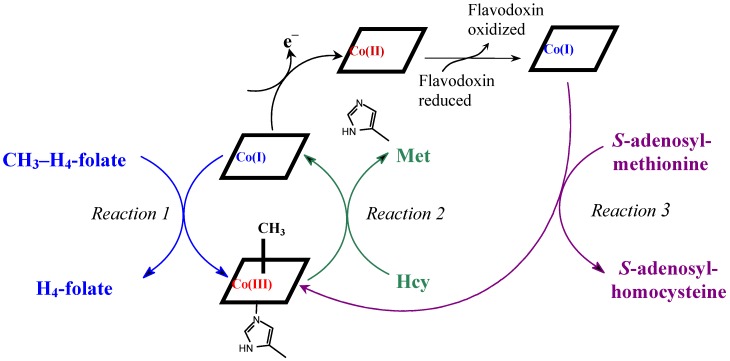
The catalytic cycle and the coenzyme reactivation in methionine synthase. Reactions 1 and 2 are involved in the catalytic cycle and reaction 3 in the cobalamin reactivation. In the reactivation reaction, it has been suggested that histidine is displaced from cobalt in cob(II)alamin as shown on the top of the drawing.

The synthesis of methionine (Met) from homocysteine (Hcy), by transfer of a methyl group from N5-methiltetrahydrofolate, is catalyzed by MetH according to the following reaction: 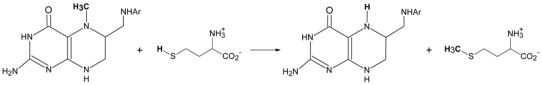


The catalytic cycle is reported in Figure 7. Cob(I)alamin, demethylates CH_3_–H_4_-folate (Figure 7, reaction 1) to form MeCbl and H_4_-folate, which transfers the methyl group to Hcy (Figure 7, reaction 2) to form methionine and regenerating cob(I)alamin. Under microaerophilic conditions, cob(I)alamin is oxidized and converted to the inactive cob(II)alamin, about once per every 2,000 turnovers, deactivating the enzyme. The reactivation occurs by reduction of cob(II)alamin by flavodoxin to cob(I)alamin and methylation by *S*-adenosyl-L-methionine (AdoMet) (Figure 7, reaction 3) to MeCbl, which is returned to the catalytic cycle. Thus, the enzyme function involves the chemistry of cobalamins in three different oxidation states and coordination numbers. The function and structure of MetH has been reviewed [6,7,8,11,28]. However, elucidation of these aspects represents an impressive piece of work and merits to be summarized here. 

**Figure 8:**
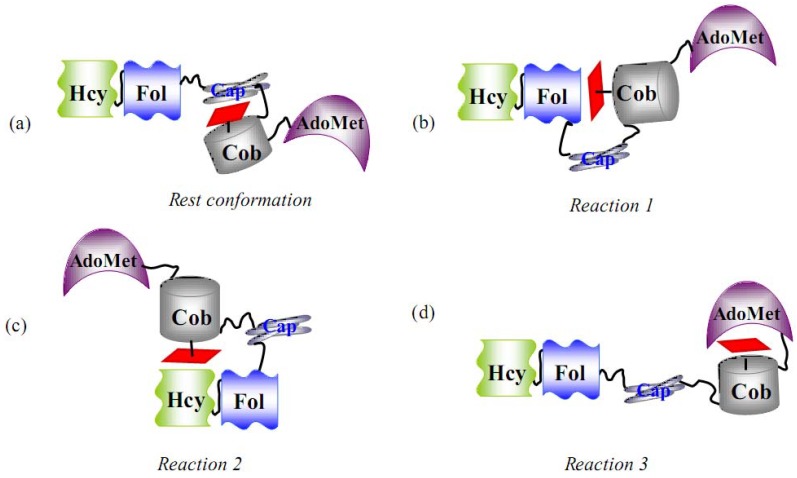
Proposed four conformational states of MetH in solution: (a) unknown conformation in the rest state; (b) conformation for reaction 1 in Figure 7; (c) conformation for reaction 2 in Figure 7; (d) conformation for reaction 3 in Figure 7. The four modules are shown in green (Hcy), in blue (Fol), in gray (Cap-Cob) and in magenta (Adomet) respectively. The red rectangle represents the cobalamin and the vertical line in a), (b) and (c) represents its binding to the histidine of the Cob domain. In d) the position of the vertical line indicates that the histidine is displaced from Co.

The reactions, sketched in Figure 7, are performed by the MetH enzyme from *Homo sapiens* and *Escherichia coli* (136 kDa), which consists of the four sequential modules shown in Figure 8a. The structure of full-length MetH is not available. However, a strategy involving expression and/or isolation of the modular fragments furnished structural insight and allowed delineation not only of the structural organization, but also of several functional aspects of MetH, which could not have been obtained from the knowledge of the three-dimensional structure of the full MetH alone. 

**Figure 9:**
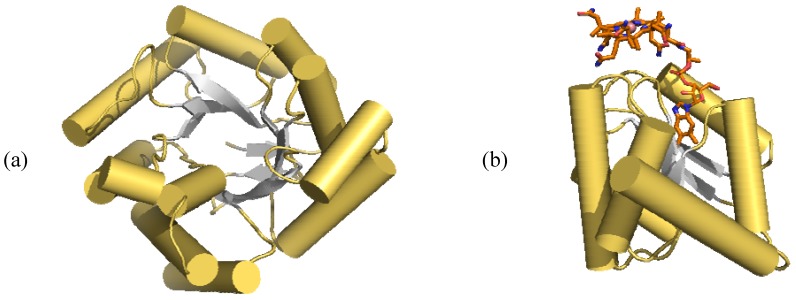
The Rossmann fold found in: (a) Fol domain (PDB 1Q8J); (b) Cob domain of (PDB 3BUL) MetH from *Escherichia coli*. The His domain is similar to that of Fol.

The two N-terminal domains, indicated as Hcy and Fol in Figure 8, are the homocysteine-binding domain and CH_3_-H_4_Folate-binding domain, respectively [57,58]. Both domains show the (β/α)_8_ TIM barrel (found in triosephosphateisomerase) that is generally observed for substrate-binding domains of B_12_ enzymes (Figure 9a). The two barrels, whose axes are oriented perpendicularly to each other, are firmly associated and resist proteolysis. The Hcy binding domain contains a zinc ion as essential cofactor, which binds three Cys and one Asn residues in the absence of homocysteine. When homocysteine binds the Hcy domain, the Asn residue is displaced by homocysteine (as thiolate) to complete the tetrahedral four-S coordination about zinc [58]. 

**Figure 10:**
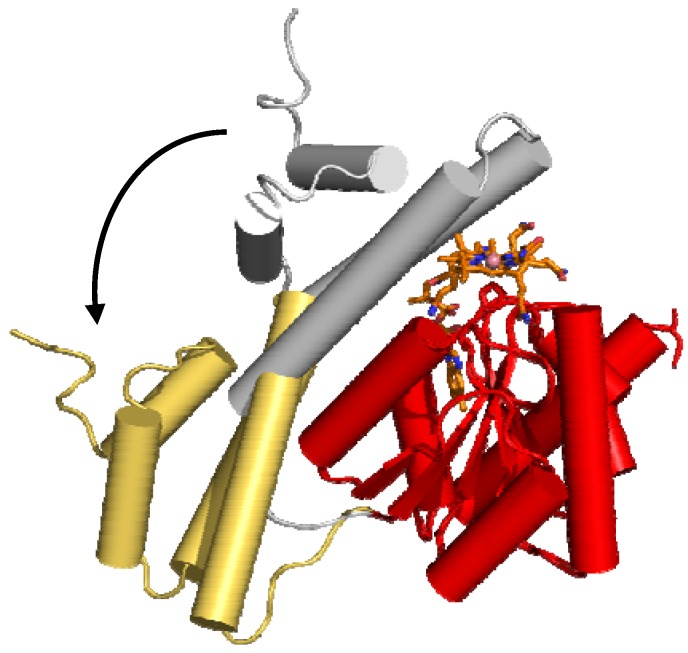
The large movement of the Cap sub-domain of methionine synthase from the inactive Cap-Cob domain (in silver cartoon, PDB 1K7Y) to the active form (gold cartoon, PDB 1BMT). The Cob sub-domain with bonded cobalamin (in stick) is shown by red cartoons.

The third module, Cap-Cob, is the binding site of cobalamin, where cobalamin is sandwiched between the Cap and Cob sub-domains in its base-off form. The Cap sub-domain, a four-helix bundle, covers the β face of the cobalamin and is connected by a linker to the Cob sub-domain, which has a α/β Rossmann motif [55], usually found as Cbl-binding site in other enzymes (Figure 9b). Cobalt is coordinated by a histidine residue of the Cob sub-domain on the corrin α face and the displaced nucleotide is embedded into the latter sub-domain. Thus, Cbl is bound to the protein site in the base-off/His-on mode. This Cbl binding mode is associated with the consensus sequence Asp-X-His-XX-Gly, where His coordinates Co [59]. However, when the Cap-Cob domain enters in contact with the substrate domains, the Cap subdomain is displaced by about 25 Å and rotated by about 60° in order to open the cobalamin β face to the corresponding substrate (Figure 10) [60,61]. The fourth module, the reactivation domain, is the S-adenosyl-L-methionine binding site (AdoMet). These structural studies, together with spectroscopic investigations, which exploit differences in the UV-Vis spectra of the cobalamin in different coordination and oxidation states [62,63], has allowed the proposal that in solution there are at least the four conformations sketched in Figure 8. The enzyme should assume the three conformation b-c) in order to catalyze reactions 1-3, respectively. This implies that the enzyme must undergo large conformational changes in order to allow the binding domains of the substrates to access the cobalamin-binding domain in the three methyl transfer reactions of Figure 7. Interestingly, the finding shows that the *trans*-influence of the β axial ligand governs conformation in cobalamin-dependent methionine synthase, following the same order of *trans*-influence observed in isolated cobalamins [63] (Section 4). As found in the complex of Cbl in complex with TC, the chemistry of cobalamins is at least qualitatively conserved when they are bound to the protein (Section 5.1).

#### 6.1.2. Corrinoid-dependent methyltransferase in archaea and bacteria

In this section only a few examples of corrinoid-dependent methyltransferases will be examined, particularly those whose structure is known. Anaerobic methanogenic archaea reduce C_1_-compounds to methane [19]. Their enzymes, able to transfer the methyl group from a methyl containing substrate to ethane thiolsulphonate (coenzyme M, HS-CoM), have been very recently reviewed [8]. Those so far studied have as substrate methanol, (CH_3_)_n_H_3-n_ (n = 1,3) amines, (CH_3_)_4_N^+^ and methylthiols. The three modules of Figure 6 well represent a sketch of the arrangement and functioning of these enzymes, which have a striking similarity to the MetH behaviour. However, differently from MetH, each module is represented by a different protein, complexed with the other two in a BCA protein complex, where C is the B_12_ binding protein and A and B represent the acceptor and donor proteins, respectively. The B module catalyzes the transfer of the CH_3_ group to Co(I) in the central module and the A module catalyzes the CH_3_ transfer from CH_3_-corrinoid to coenzyme M to form CH_3_-SCoM. The most characterized enzyme is MtaABC, which catalyze the following reaction:

CH_3_OH + HS-CoM → CH_3_-S-CoM + H_2_O

The sequenced corrinoid-binding domain (the central module of Figure 6) of those enzymes shows homology with the cobalamin-binding domain of MetH, including the characteristic Asp-X-His-X-X-Gly motif containing the coordinated His. The presence of this motif is indicative of a corrinoid binding to the protein in the base-off/His-on mode. However, no significant homology is found between the substrate binding domain and the Hcy domain in methionine synthase. In spite of the latter observation, the X-ray crystal structure of the MtaBC moiety of the MtaABC enzyme from *Methanosarcina barkeri* (the only X-ray structure thus far available for these enzymes) has shown [64] that the methyl donor module MtaB is composed by a TIM barrel, similar to that found in the Hcy and Fol domains of Met H (Figure 9b), encircled by seven α helices. It contains the essential cofactor Zn^2+^ ion in a deep funnel-shaped pocket and a putative K^+^ ion at a distance of 3.1 Å. No methanol substrate was detected. The Zn^2+^ ion is coordinated by two Cys and one Glu residues in an incomplete tetrahedral arrangement lacking the fourth ligand. The MtaC module, the 5-hydroxy-benzimidazolylcobamine binding protein, contains the α/β Rossmann motif connected by a linker to a four-helix bundle. The latter is displaced from the corrinoid, to allow juxtaposition of MtaB to the Rossmann domain in the folding shown in Figure 10, with the helical domain in gold. The corrinoid binds in the base-off/His-on mode, with His belonging to the sequence Asp-X-His-X-X-Gly. No axial ligand was detected on the corrin β face, so that Co(III) was proposed to be reduced to pentacoordinated Co(II) during X-ray exposure. The two MtaB and MtaC domains are bound in such a way as to position the Zn^2+^ ion above the cobalt at 7.7 Å and to define the binding site of methanol, which was suggested to coordinate Zn^2+^ in the fourth coordination position, as well as the putative K^+^ ions.

**Figure 11:**
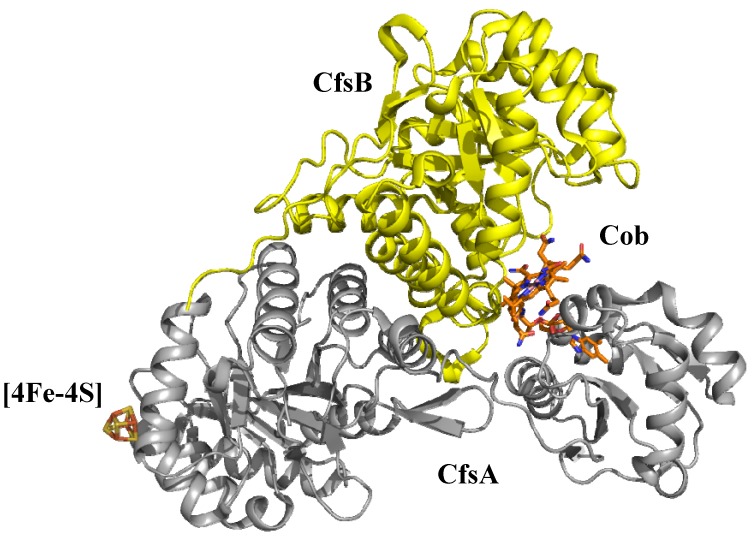
Ribbon representation of CoFeSP from C. hydrogenoformans (PDB 2H9A). The CfsB domain is shown in yellow cartoon and CfsA in silver cartoon. Cobalamin and iron-sulphur cluster are shown as sticks.

Anaerobic acetogenic bacteria and archaea produce acetate employing the Wood-Ljungdahl pathway [18]. In the final step of the pathway, acetyl-coenzymeA is synthesized from a methyl cation, CO and CoA in a reaction catalyzed by the Ni-Fe-containing acyl-CoA synthase (ACS). In the corrinoid iron-sulphur protein (CoFeSP), Co(I) abstracts the methyl group from methyltetrahydrofolate bonded in the donor module of Figure 6, to form the Co(III)-CH_3_ bond in the central module, and transfers it to the Ni centre of ACS. Since Co(I) in the central module undergoes oxidation to Co(II) about once every hundred turnovers, the low-potential iron-sulphur centre provides the reductive reactivation of the inactive cob(II)amide, as occurs in the AdoMet domain of MetH, but without the need of the methyl transfer from AdoMet. In addition, methanogens express CoFeSP proteins as part of a more complex multi-enzyme system, which catalyzes the reverse reaction of acetyl-CoA synthesis, transferring the methyl group of the latter to analogues of tetrahydrofolate and producing CO [65]. Several CoFeSP proteins from both bacteria and archaea have been isolated and characterized. They are heterodimers with a large and a small subunit and contain two cofactors. In CoFeSP from *Moorella thermoacetica* both subunits are necessary for tight binding of the 5-methoxybenzimidazolylcobamide, which is bonded to the protein in base-off form in all the Co oxidation states: neither the benzimidazole base nor a histidine residue coordinates to Co. This is a striking property of acetogenic and methanogenic CoFeSP enzymes, which also lack the consensus sequence associated with the base-off/His-on binding mode of the corrinoids. Removal of the α axial ligand is thought to contribute in enhancing the methyl acceptor and donor properties of the corrinoid in CoFeSP proteins [18]. The other cofactor is a [4Fe-4S]^n+^ (n = 1, 2) cluster, which reduces the inactive oxidized Co(II) to Co(I). The only thus far crystallographically characterized CoFeSP protein is that from *Carboxydothermus hydrogenoformans* [66]. The X-ray structure shows (Figure 11) that the heterodimer comprises two subunits, the large CfsA and the small CfsB units. 

The CfsA subunit consists of three domains: the N-terminal domain binds the [4Fe-4S] cluster, the middle one displays a (β,α)_8_ TIM barrel and the C-terminal domain shows a Rossmann α/β motif. Similarly to the cobalamin binding in MetH, the C-terminal domain surrounds the displaced 5,6-dimethyl benzimidazole nucleotide moiety and interacts with the α side of the cobalamin in a base-off form, with Co coordinated by a water molecule on the β side. In contrast with the binding of the cobalamin in the Cap-Cob domain of MetH, no aminoacid residue coordinates Co on the α side, so that the Co is pentacoordinated in an oxidation state +2, and the cobalamin binding occurs in a base-off/His-off mode. In addition, the α side interacts with an α-helix (cap-helix), which is at van der Waals distances. The CfsB subunit displays a single domain folded in a (β,α)_8_ TIM barrel, which interacts with the β side of the cobalamin. Thus the corrin macrocycle is sandwiched between CfsB and the C-terminal domain of CfsA. The two (β,α)_8_ TIM barrels in CoFeSP have a fold similar to that of the Fol domain of MetH. The overall arrangement found in the crystal structure was suggested to represent a resting state. Therefore, the Cbl binding domain of CfsA was suggested to be the mobile element able to interact with the TIM barrels in CfsA and CfsB for donating and accepting the methyl group [66]. 

### 6.2. AdoCbl-dependent enzymes

The chemistry underlying the catalysis of AdoCbl-dependent enzymes has attracted great interest among inorganic and organic chemists due to its fascinating features. In the past, during the B_12_ chemistry and biochemistry era, this produced an enormous amount of work on Cbl [4,5] and their simple models, namely the cobaloximes [24,67], However, the attention of chemists, biochemists and enzymologists is now mainly focused on the study of enzymes. Very recently, the properties [6,8] and the structure [11,28] of AdoCbl-dependent enzymes have been reviewed in detail. Therefore, in this section we will summarize essentially their main features, while the above cited reviews can be addressed for more detailed aspects. 

As stressed in Section 2, AdoCbl-dependent enzymes, mutases and eliminases catalyze the 1,2 shift of an H atom and a group on adjacent carbon atoms. According to Toraya [68], these enzymes can be grouped in three classes as shown in Table 3, where the catalyzed reaction for each known enzyme is indicated. Mutases catalyzing C skeleton rearrangement, belong to class I; eliminases catalyzing heteroatom elimination reactions, belong to class II and need K^+^ as cofactor in addition to AdoCbl, even if for ethanolamine ammonia lyase there is no clear evidence of the presence of a K^+^ or NH_4_^+^ cation; aminomutases, catalyzing intramolecular NH_3_^+^ group migration, belong to class III and need as additional cofactor pyridoxal-5’-phosphate. Ribonucleotide triphosphate reductase (RNR) is included in class II, although AdoCbl in this enzyme only serves as radical generator, which however does not interact with the substrate as occurs in the other AdoCbl enzymes (see below). From the point of view of the cofactor binding mode to the apoenzyme, the three classes can be grouped into two categories. In class I mutases and class III aminomutases, AdoCbl binds to the protein in base-off/His-on mode, and all have the consensus sequence, Asp-X-His-X-X-Gly, whose His coordinates to Co [59]. In class II, including RNR, AdoCbl binds the protein in the base-on form. From the structural point of view, in mutases and eliminases, but not in RNR, the AdoCbl binding domain has always an α,β Rossmann fold (Figure 9b) and the substrate is bonded in the (α,β)_8_ TIM barrel (Figure 9a), as in the other B_12_ dependent enzymes.

All the bacterial isomerases are heterodimeric (or heterotetrameric) with only one of their sub-unit binding the cofactor, except MGM and MMCM from *E. coli* (Table 3). The latter two isomerases and mammalian MMCM are homodimeric with two cofactors for each subunit [69]. Eliminases, such as DD and GD, are homotrimers containing two cofactor molecules, whereas EAL is heterohexameric [8,70]. The X-ray structures of MMCM from *P. shermanii*, GLM from *C. chlochearium*, LAM from *C. stricklandii*, DD from *K. oxytoca* and DD from *K. pneumoniae*, in several cases also in the apo form, with and without the substrate and/or reconstituted with other XCbl, have been reported [11,28,69].

**Figure 12:**
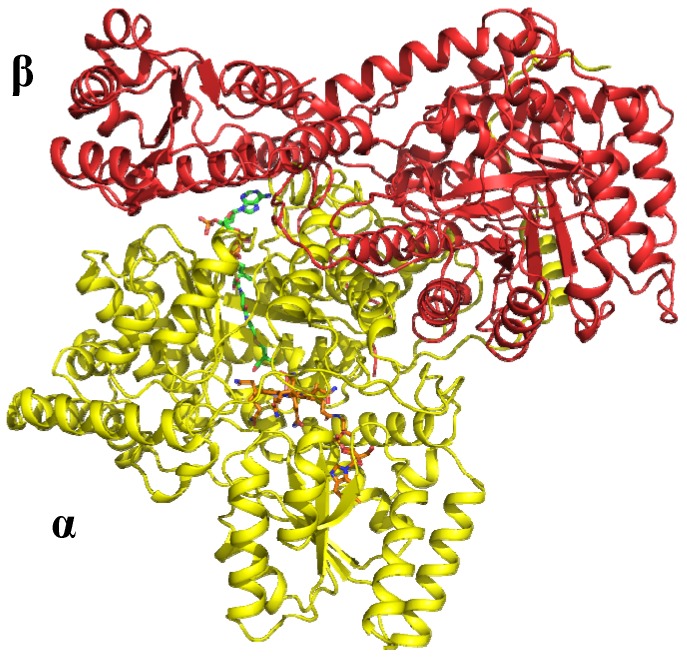
Ribbon representation of Methylmalonyl-CoA Mutase, MMCA (PDB 7REQ). The monomers α and β are shown in yellow and silver cartoon, respectively. Cobalamin is shown by orange sticks, while 2-carboxypropyl-CoA is represented with green sticks.

The X-ray structure of methylmalonyl-CoA mutase (MMCM) from *P. shermanii* was the first structural determination of a complete Cbl enzyme [56] and we would like to summarize its main structural features (Figure 12). The α and β chains of the αβ heterodimer of 149 kDa have similar folding. However, a modified substrate (desulpho-CoA) and AdoCbl bind only the α chain, in the (α,β)_8_ TIM barrel and α,β Rossmann domains, respectively. In contrast, the human MMCM is an α_2_ homodimer of 150 kDa, each α subunit having one substrate-binding site and one Cob domain. No electron density for the adenosyl group was detected, suggesting that the coenzyme is present in the reduced cob(II)alamin base-off/His-on form. On the contrary, the substrate-free holoenzyme structure clearly showed [11] the intact AdoCbl, where the adenine moiety stacks with the side chain of the Tyr A89 residue [see below].

The comparison of the latter structure to that with the desulpho-CoA substrate also indicates that large conformational changes occur when the substrate binds the protein. Particularly, the (α,β)_8_ TIM barrel is split apart and the active site is accessible to solvent in the substrate-free structure. When the substrate binds, the barrel encloses the substrate and the active site is completely buried. Finally, the structure of MMCM with a methylmalonylCoA/succinylCoA mixture (50%) in the (α,β)_8_ TIM barrel showed electron density for partial occupancy of a 5′-deoxyadenosine not coordinated to cobalt and with its 5′-carbon atom near the substrate. This structural result supports the hypothesis that the entrance of the substrate significantly contributes to the enhancement of the Co-C bond cleavage. In fact, the binding of the substrate induces severe conformational changes in the protein [11], with the closing up of the TIM barrel about the substrate, which should provoke further activation of the Co-C bond, in addition to that resulting from the binding of AdoCbl to the protein scaffold.

**Figure 13:**
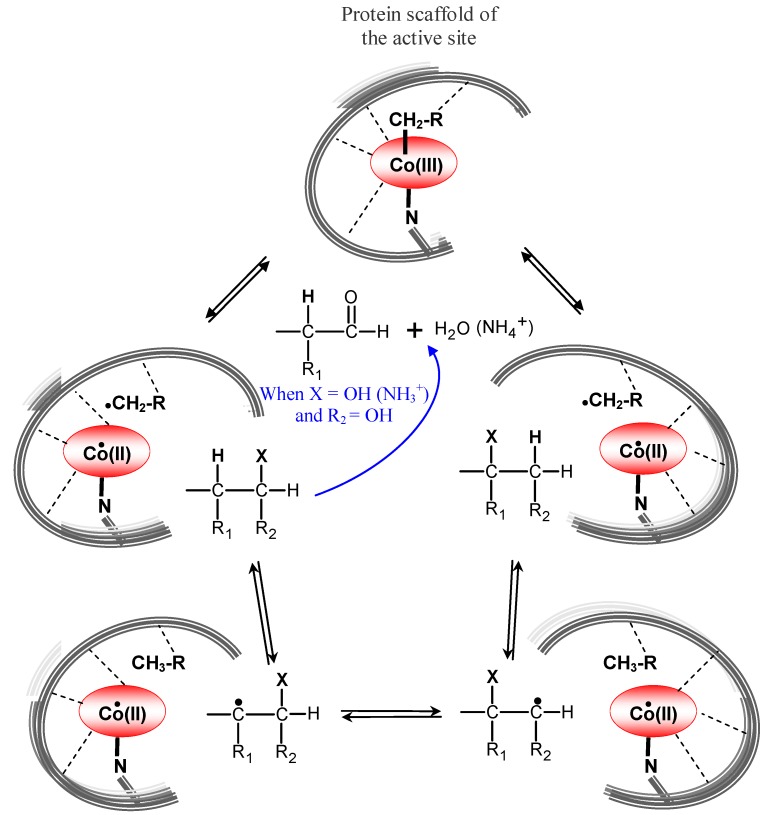
The catalytic cycle in AdoCbl enzymes (CH_2_R = Ado). The blue arrow indicates the final product in eliminases. The broken lines into the active site represent a sketch of the H-bonds between the cofactor and the protein aminoacid residues.

The basic enzymatic mechanism which applies to all AdoCbl-dependent enzymes, except RNR, was separately elucidated by Abeles [71] and Arigoni [72] and it is sketched in Figure 13. After binding of the substrate, the reaction starts with the Co-C bond homolysis and formation of the Ado• (RCH_2_• in Figure 13) and cob(II)alamin radicals. The RCH_2_• radical abstracts an H atom from the substrate to produce a substrate radical and 5′-deoxyadenosine (RCH_3_). The substrate radical next rearranges into the product radical, by exchanging H and X on adjacent carbon atoms (Figure 13). The product radical then abstracts an H atom from 5′-deoxyadenosine to form the product, which is released, and the formed RCH_2_• radical restores the Co-Ado bond. In the case of eliminases, the product is a 1,1-dialcohol (or 1,1-aminoalcohol), which subsequently eliminates water (or ammonia) and the resulting aldehyde is released. Thus, whereas in mutases, such as MMCM, the reaction is reversible and hence the product and substrate are interchangeable, in the eliminases, such as dioldehydratase (DD), the reaction can obviously not be reversed. In RNR, the RCH_2_• radical produced by homolysis abstracts a H atom from a Cys residue generating a thiyl radical, which in turn abstracts a H atom from the substrate initiating the reductive rearrangement of ribonucleoside to 2’-deoxyribonucleoside. The X-ray structure shows [73] that the AdoCbl binds the protein in base-on form and its binding domain has no structural similarity with that of all the other B_12_ enzymes [11,28]. 

As stressed by Rowina Matthews [8], the mechanism of Figure 13 poses several challenges, some of which are just now beginning to be understood thanks to recent X-ray structural, spectroscopic and theoretical studies [6,7,8,11]. First of all, as stressed in Section 2, the means by which these enzymes catalyze the homolytic cleavage of the Co-Ado bond several orders of magnitude [6,7,8,34] faster than the free coenzyme is unknown and still remains a topic of intense research. Second, how does the H transfer from substrate to the RCH_2_• radical and from RCH_3_ to the product radical occur (Figure 13) for some enzymes in which the H must travel long distances (up to 10 Å). Recent observations [74] suggest that, in mutases the distance between cob(II)alamin and the substrate radical is consistent with the participation of the former in the rearrangement of the latter. Theoretical calculations do support this suggestion [75]. However, this is not the case of eliminases, in which that distance is too great for an involvement of cobalamin in the rearrangement. Very recently, theoretical DFT calculations have suggested that the tyrosine residue facing the Ado moiety of the cofactor in MMCM and GLM, may participate in the enzymatic process and contribute to the activation of the Co-C bond [76]. 

### 6.3. Adenosyltransferase

Adenosyltransferase is an example of enzymes where AdoCbl does not play the role of a cofactor but that of a substrate. As has been stressed in Section 5, mammals are unable to synthesize AdoCbl. Consequently, the Cbl must be adsorbed from food and transported into the mitochondria, where an ATP:corrinoid Adenosyltransferase (ACA) catalyzes the transfer of the 5’-deoxyadenosyl moiety of ATP to the upper axial position of Cbl to generate AdoCbl (see the top side of Figure 3). The AdoCbl is then transferred as cofactor to the MMCM. Malfunction of human ACA can lead to disorders, such as methylmalonylaciduria, a rare but fatal disease [77]. In order to obtain AdoCbl, Co(III) in the originally ingested cobalamin, probably as H_2_O(HO)Cbl, must be reduced to cob(II)alamin, which in turn is reduced to cob(I)alamin, which, finally, attacks the 5’-carbon atom of ATP, generating AdoCbl. The reducing intracellular environment easily allows the Co(III)/Co(II) reduction, which has a reduction potential of 200 mV. However it is not low enough to drive the reduction of cob(II)alamin to cob(I)alamin, which has a reduction potential of -600 mV. Spectroscopic and computational studies support the hypothesis that ACA enzymes bind cob(II)alamin and convert it, in the presence of ATP, to the rare Co(II) base-off/His-off species. The latter species should have a higher reduction potential and can be more easily reduced to cob(I)alamin [78]. There are three structurally distinct families of ACA: CobA, EutT and PduO. Human ACA belongs to the PduO-like family. No X-ray structure of EutT species has so far been reported, whereas the structure of a human-type PduO from *Homo sapiens* [79] and *Lactobacillus reuteri* [80] has been reported in complex with ATP. This complex exhibits very similar structure, but with an architecture quite different from that of CobA complexed with ATP and HOCbl [11,28]. Finally, the structure of a human-type ACA in complex with Mg^2+^ATP and cob(II)alamin was reported and confirmed the previous suggestion that cob(II)alamin is four coordinated, binding the protein in the base-off/His-off form [81]. More recently, the enzymatic activity and the X-ray structure of the mutants of human-type PduO, PduO^F112A^ and PduO^F112H^, have been reported [82]. This study indicates that the residue Phe 112 is critical in the displacement of nucleotide benzimidazole to form the tetracoordinate cob(II)alamin. In fact, the catalytic efficiency was found to be reduced by 80% in PduO^F112A^ and to decrease four orders of magnitude in PduO^F112H^ with respect to that of the wild-type enzyme. The explanation of the enormous decrease in activity has been ascribed to the formation of a base-on cob(II)alamin in both mutants: the structural analysis indicated that the cob(II)alamin is present in a pentacoordinated base-on form in the PduO^F112A^ mutant, whereas it is in a hexacoordinate base-on/His-on form in the PduO^F112H^ mutant.

## 7. B_12_ Bioconjugates

For many years, the transportation pathway and cellular uptake of vitamin B_12_ in humans has suggested the use of Cbl to enhance the oral bioavailability of tethered peptides, proteins and drugs with poor solubility and low intestinal absorption. This aspect has very recently been reviewed, with particular emphasis on peptide and protein delivery [83]. Recent progress includes the delivery of EPO, hormones and, more recently, insulin [84]. 

Furthermore, since fast proliferating cell types require higher amounts of vitamin B_12_ than normal cells, another way to exploit B_12_ cellular uptake was followed in designing and testing bioconjugates, able to deliver radio and NMR imaging agents or cytotoxic agents to cancer cells. Specifically, the functionalized bioconjugates can be employed as “Trojan horses” to carry the antitumoural or diagnostic label into the cell [85]. This approach is based on up-regulation of the TC-Cbl receptors on the cell surface of tumour cells in the DNA replication phase and possibly by higher turnover of the receptors [15]. 

**Figure 14:**
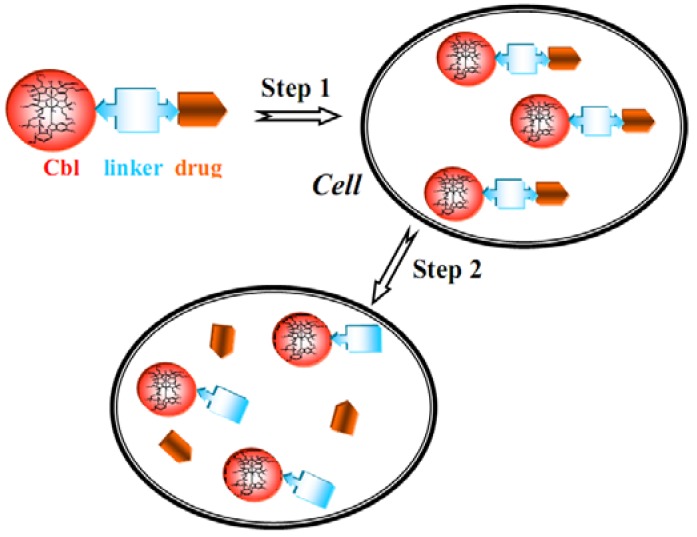
Sketch of a B_12_ bioconjugate and its functioning for imaging (step 1 and 2) and for antitumour activity (step 2). In step 1 the inactive labelled complex enters the tumour cell. In step 2 the drug is released by cleavage of its binding to the linker, by different way (pH, enzymatic process, *etc*.).

Bioconjugates may be prepared with relative ease, exploiting the various functionalities of cobalamins (Figure 1) that can covalently bind the suitably designed imaging or cytotoxic labels [48]. In principle, conjugation may occurs by: i) reaction with the carboxylic groups prepared by hydrolysis of the peripheral corrin amide side chains; ii) by esterification at cobalamin’s ribosyl 2’-OH and 5’-OH (OR7 and OR8, respectively in Figure 1b); iii) by reductive alkylation at the β axial position of the Co(III) centre; iv) by coordination to the N atom of the β axial CN group in CNCbl. The most exploited positions are the corrin *e* side chain and the 5’-OH ribosyl group, in addition to the Co β axial position (Figure 1a). The X-ray structure of the Cbl complexes with TC [13] and IF [14] indicated that the latter two positions were also those which appear most promising to not interfere with the binding of the bioconjugate to the transport proteins. Cbl and the bonded group in the bioconjugate can be coupled together directly or distanced by a spacer unit. The covalent binding of the label can easily be broken. The cleavage of this bond is important when a cytotoxic drug should be released into the cell (Figure 14). 

**Figure 15:**
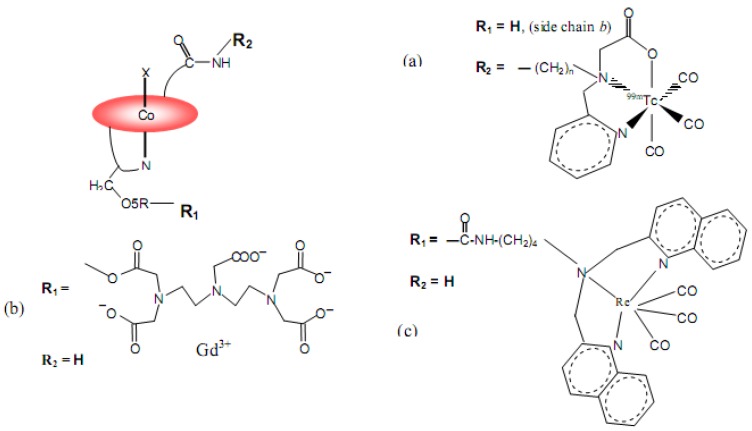
Some more recent B_12_ bioconjugates.

Work in this field has been reviewed recently [28,48]. A study has shown that a radiolabeled bioconjugate (Figure 15a, n = 2-6) binds to haptocorrin and is able to target several tumour cells, avoiding undesirable accumulation in organs, such as kidneys, which are TC-Cbl storage sites [16]. In this case the bioconjugates, with appropriate spacer length (n < 4), do not bind to TC, but do to HC and IF. This pattern of interaction was not expected on the basis of experimental [12] and theoretical data [45,47]. 

Very recently, two bioconjugates, namely CNCbl-DTPA-Gd (DTPA= diethylenetriamine-*N,N,N′,N″,N‴*-pentaacetic acid) (Figure 15b) and the homologous CNCbl-TTHA-Gd (TTHA = triethylenetetramine-*N,N,N′,N″,N‴,N‴*-hexaacetic acid), have been synthesized and characterized [86]. The former complex has been also structurally characterized by X-ray crystallography. They have been shown to maintain binding to TC close to that of CNCbl. However, it was found that CNCbl-DTPA-Gd, but not the more stable CNCbl-TTHA-Gd, is able to release a considerable amount of free Gd^3+^ ions *in vitro*. The internalization of the toxic Gd^3+^ ions into the human myelogenous leukaemia K562 cells at the cytotoxic level causes a marked decrease in the tumour cell viability. Therefore, the ability of CNCbl-DTPA-Gd to deliver toxic Gd^3+^ ions to tumour cells opens a new perspective in the development of B_12_ bioconjugates that hide and transport such toxic entities, although the corresponding mechanism is not yet understood. The Re(I) B_12_ bioconjugate of Figure 15c in complex with IF, has been shown to be internalized into the choriocarcinoma BeWo cell line in an IF cubilin mediated fashion *via* the siRNA transfection experiment [87]. This study has shown the potential of the IF receptor, cubilin, as a new target for delivery of B_12_ conjugates into tumour cells. All these recent findings, together with previously obtained data, have opened further possibilities to find B_12_ conjugates for cancer diagnostics and treatment. However, the mechanism of internalization in some cases requires further studies to be fully understood. 

## 8. Conclusions

This review has outlined the enormous impact of structural studies on the development of B_12_ chemistry and biochemistry and, more recently, to B_12_ enzymology. A lot of experimental and theoretical work has been done to assess the influence of cobalamin’s axial ligands and the protein environment on the organometallic chemistry of cobalt and its stability in a given oxidation state. Based on the B_12_ enzyme and protein structures, available to date, it appears that regarding transport proteins, Cbl prefers to bind in base-on form. Instead, the dominating form when B_12_ binds to enzymes is the base-off one. The reason behind this difference and in particular the function of the histidine substitution for dimethylbenzimidazole in some structures with Cbl in base-off form is still a matter of investigation. The way by which AdoCbl enzymes catalyze the Co-C bond homolysis 10^12^ times faster than the free coenzymes is still unclear. Analogously, the intracellular processes leading from endocytosed TC-Cbl to the formation of AdoCbl in mitochondria as well as of MeCbl in the cytosol are not yet fully understood. More contributions are certainly expected in elucidating the role of B_12_ in the mechanism of carbon dioxide fixation in anaerobic acetogenic bacteria and methanogenic archaea, as well as in the reductive dehalogenation of chlorinated hydrocarbons. The very recently reported X-ray structure of the complex of intrincsic factor with its receptor has opened the way to the understanding of the B_12_ internalization on a structural basis. Further studies are expected to find new vitamin B_12_ bioconjugates as imaging agents for diagnosis of tumour cell or as therapeutic drugs exploiting conjugated groups with growth blocking or cytotoxic properties.

## Figures and Tables

**Table 1:** Relevant structural aspects of AdoCbl and MeCbl in base-on form (data from refs. [6] and [24]).

	**d_Co-C_^a^**	**d_Co-NB3_^a^**	**Co-C-C^b^**	**ν_Co-C_^c^**	**BDE^d^**
**AdoCbl**	2.033(4)	2.237(3)	123.4(2)	430	125 ± 8
**MeCbl**	1.979(4)	2.162(4)	-	506	155 ± 13

^a^ Axial distances (Å) in Figure 1. Estimated standard deviation in parentheses; ^b^ The Co-CH2-R angle (°) in Ado-Cbl. Estimated standard deviation in parentheses; ^c^ Co-C stretching frequencies (cm^-1^); ^d^ BDE = Co-C bond dissociation enthalpy (kJ/mol) from kinetic methods in the range 80-110 °C. More recent data from calorimetric methods in the range 10-30 °C gave 129 ± 17 kJ/mol and 163 ± 21 for AdoCbl and MeCbl, respectively. The BDE of MeCbl in base-off form is evaluated to be 176 ± 23 [27].

**Table 2:** Values of p*K*_base-off_ (at 25 °C), *K*_co_^a^ and Co-NB3 distances (Å)^b^ for XCbl’s.

X	p*K*_base-off_^a^	*K_Co_*	*ΔG^0^_Co_ (kJ/mol)*	*Co-NB3^b^*
**NO**	5.1	1.9	-1.59	2.349 (2)^c^
**CH_3_CH_2_**	4.16	2.00·10	-7.42	2.232 (1)^d^
**CH_3_CH_2_CH_2_**	4.10	2.37·10	-7.84	/
**Ado**	3.67	7.25·10	-10.6	2.237 (3)
**CH_3_**	2.90	4.52·10^2^	-15.1	2.162 (4)
**AdoPr**	3.31	1.77·10^2^	-12.8	2.212 (8)
**CF_3_CH_2_**	2.60	9.23·10^2^	-16.9	/
**CH_2_=CH**	2.4	1.5·10^3^	-18	2.165 (6)
**cis ClCH=CH**	2.3	1.8·10^3^	-19	2.144 (5)
**CF_2_H**	2.15	2.60·10^3^	-19.5	2.187 (7)
**NCCH_2_**	1.81	5.62·10^3^	-21.4	/
**CF_3_**	1.44	1.32·10^4^	-23.5	2.05 (1)
**CN**	0.10	2.88·10^5^	-31.1	2.041 (3)
**H_2_O**	-2.13	4.90·10^7^	-43.9	1.925 (2)

^a^ Ref. [6]; ^b^ Ref. [24]; ^c^ Ref. [30]; ^d^ Ref. [31].

**Table 3:** Reactions catalyzed by cobalamin dependent enzymes. GLM = Glutamate mutase; MGM = 2-Methyleneglutarate Mutase; MMCM = Methylmalonyl-CoenzymeA mutase; IBCM = i-Butyryl-CoenzymeA mutase; DD = 1,2-Diol dehydratase; GD = Glyceroldehydratase; EAL = Ethanolamine ammonia lyase; LAM = L-β-Lysine 5,6-aminomutase; OAM = D-Ornithine 4,5-aminomutase.

	*X*	*R*	*Enzyme*
**Class I**			
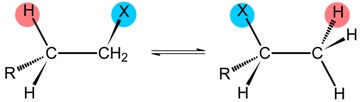	-CH(NH_3_^+^)CO_2_^−^	CO_2_^−^	***GLM***
	-C(CO_2_^−^)=CH_2_	CO_2_^−^	***MGM***
	COSCoA	CO_2_^−^	***MMCM***
	COSCoA	H	***IBCM***
**Class II**		***R***	***Enzyme***
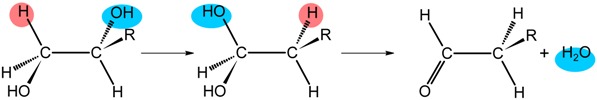		H, CH_3_, CH_2_OH	***DD*** ***GD***
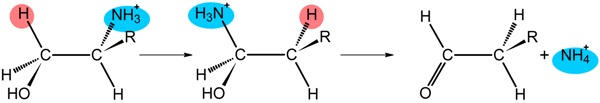		H, CH_3_	***EAL***
**Class III**	***R***		***Enzyme***
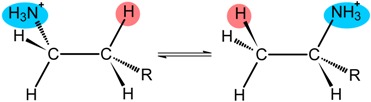	^−^O_2_C(NH_3_^+^)HC(CH_2_)_2_-		***LAM***
	^−^O_2_C(NH_3_^+^)HCCH_2_-		***OAM***
